# *Nannochloropsis* as an Emerging Algal Chassis for Light-Driven Synthesis of Lipids and High-Value Products

**DOI:** 10.3390/md22020054

**Published:** 2024-01-24

**Authors:** Ying Ye, Meijing Liu, Lihua Yu, Han Sun, Jin Liu

**Affiliations:** 1 Laboratory for Algae Biotechnology & Innovation, College of Engineering, Peking University, Beijing 100871, China; yeyingk@foxmail.com (Y.Y.); iceandcandy@163.com (M.L.); liwa1988@stu.pku.edu.cn (L.Y.); 2 Key Laboratory of Poyang Lake Environment and Resource Utilization, Ministry of Education, Center for Algae Innovation & Engineering Research, School of Resources and Environment, Nanchang University, Nanchang 330031, China; 3 Institute for Advanced Study, Shenzhen University, Shenzhen 518060, China

**Keywords:** *Nannochloropsis*, biofuels, synthetic biology, pigments, genetic manipulation

## Abstract

In light of the escalating global energy crisis, microalgae have emerged as highly promising producers of biofuel and high-value products. Among these microalgae, *Nannochloropsis* has received significant attention due to its capacity to generate not only triacylglycerol (TAG) but also eicosapentaenoic acid (EPA) and valuable carotenoids. Recent advancements in genetic tools and the field of synthetic biology have revolutionized *Nannochloropsis* into a powerful biofactory. This comprehensive review provides an initial overview of the current state of cultivation and utilization of the *Nannochloropsis* genus. Subsequently, our review examines the metabolic pathways governing lipids and carotenoids, emphasizing strategies to enhance oil production and optimize carbon flux redirection toward target products. Additionally, we summarize the utilization of advanced genetic manipulation techniques in *Nannochloropsis*. Together, the insights presented in this review highlight the immense potential of *Nannochloropsis* as a valuable model for biofuels and synthetic biology. By effectively integrating genetic tools and metabolic engineering, the realization of this potential becomes increasingly feasible.

## 1. Introduction

Up until now, fossil-derived fuels have served as the main global energy sources. The ever-increasing energy demand, depleted reserves of fossil fuels, and environmental concerns associated with fossil fuel burning, however, have led to the exploration of alternative energy that is green, renewable, and sustainable. Biomass-derived oils have a similar structure to fossil fuels, are energy rich, and represent important sources for making biodiesel. Currently, plant oils contribute to the major feedstocks for biodiesel production. Biodiesel from plants, however, is a long way from meeting the existing demand and will cause the conflict of food versus fuel. Algae has gradually come to people’s attention because of its advantages of fast growth, no competition with grain, and higher oil production potential than plants [1,2]. However, it remains economically infeasible for algae to serve as a separate biofuel producer. Combining oil production with high-value co-products using synthetic biology is a solution.

*Nannochloropsis*, a genus of Eustigmatophyceae microalgae, holds immense promise due to its unique attributes, including rapid growth, a high lipid content, and the ability to thrive in diverse environmental conditions; these characteristics make it an ideal candidate for producing oils, omega-3 fatty acids, and a wide array of high-value compounds [3]. *Nannochloropsis*, with its lipid content exceeding 30% of its dry weight, emerges as a highly promising feedstock for biodiesel production. In a study, the type 1 diacylglycerol acyltransferase (DGAT) gene derived from *Arabidopsis thaliana* (AtDGAT) was heterologously expressed in *Nannochloropsis oceanica* using electroporation. In comparison to the wild-type *N. oceanica*, the overexpression of AtDGAT resulted in a significant increase in C16 (C16:0 + C16:1) levels and a slight decrease in C18 (C18:0 + C18:1), providing novel insights into the genetic engineering of oleaginous microalgae [4]. In addition, there have been many studies exploring *Nannochloropsis* as feed for Pacific white shrimp, Atlantic salmon, kuruma shrimp, and European sea bass [5,6]. Adissin et al. [7] used algal powder to take the place of fish oil and finally improved shrimp growth. More interestingly, this strategy changed the fatty acid profile of kuruma shrimp, with a big increase in polyunsaturated fatty acids (PUFAs). *Nannochloropsis* can be used in food as well. Lafarga et al. [8] tried to add 2.0% *Nannochloropsis* to functional breads and performed an evaluation of their physicochemical properties, organoleptic properties, and nutritional properties. The new breads with a high amount of bioaccessible polyphenols showed a highly improved antioxidant capacity. Additionally, the non-living cells of *N. oceanica* can be effective adsorbents for treating textile wastewater containing azo dyes [9]. The bioactive extracts of *Nannochloropsis* are interesting targets in the medical and cosmetic industries. Khattib et al. [10] isolated an active compound of lyso-diacylglyceryl-N,N,N-trimethyl-homoserine (lyso-DGTS) from *Nannochloropsis*, which can interact with high-density lipoprotein to improve its anti-atherosclerosis abilities. Moreover, violaxanthin has significant skin-protective activity against induced oxidative stress, such as UVB-mediated cellular damage [11]. Due to the high abundance of violaxanthin (32% of total carotenoids), *Nannochloropsis* can be used as a functional ingredient in cosmetic formulations [11]. It has been found that *Nannochloropsis* produces high levels of vitamin D_3_ naturally, which is not common in other algae [12].

Synthetic biology, with its genetic and metabolic manipulative prowess, allows for the fine tuning of *Nannochloropsis* to optimize the production of lipids, proteins, carotenoids, and other high-value compounds [13,14]. Synthetic biology provides the essential toolbox for reprogramming *Nannochloropsis* at the genetic level. It involves a spectrum of tools, such as the revolutionary CRISPR-Cas9 gene-editing system, custom-designed promoters, and metabolic pathway engineering [15,16]. These tools empower researchers to tailor *Nannochloropsis* strains for trait improvements, including but not restricted to growth, oils, omega-3 PUFAs, and high-value carotenoids.

This review aims to provide a comprehensive overview of the current state of *Nannochloropsis*-based biorefining driven by synthetic biology [13]. It will delve into the taxonomy and growth characteristics of *Nannochloropsis*, the fundamental principles of synthetic biology, the tools and techniques employed in genetic and metabolic engineering, and the various biorefining strategies being pursued. Furthermore, it will explore the challenges faced in scaling up *Nannochloropsis*-based biorefining processes, address environmental considerations and sustainability, and touch upon the regulatory and ethical aspects governing this rapidly advancing field.

## 2. Growth Physiology of *Nannochloropsis*

*Nannochloropsis* is a genus of unicellular and non-motile marine microalgae classified within the phylum Heterokontophyta, the class Eustigmatophyceae, and the family Eustigmataceae. This genus was initially defined by Hibberd and comprises six well-documented species: *Nannochloropsis gaditana*, *Nannochloropsis granulata*, *N. oceanica*, *Nannochloropsis oculata*, *Nannochloropsis salina*, and *Nannochloropsis australis* [17,18]. *Nannochloropsis* is characterized by small, unicellular, non-motile, and spherical or ovoid cells. It is a single-celled organism, typically ranging from 2 to 8 μm in size, constituting an entire living entity within a solitary cell. This distinction sets it apart from multicellular organisms, which are formed through collaboration between multiple cells [3]. The cells are encased within a unique cell-wall structure composed of complex polysaccharides, offering protection and stability. The inner layer of the cell wall in *Nannochloropsis* displays a porous structure with a delicate fibrous substructure and support structures linking this layer to the plasma membrane [19]. In certain *Nannochloropsis* species, small amounts of additional sugars (such as rhamnose, mannose, ribose, xylose, fucose, and galactose) may be present [20]. The ultrastructure of *Nannochloropsis* cells, including the organization of chloroplasts and other organelles, has been studied in great detail using electron microscopy techniques, shedding light on the intricacies of its cellular architecture [3].

*Nannochloropsis* exhibits rapid growth rates under optimal conditions, making it an attractive candidate for biotechnological purposes. Its growth physiology encompasses several key factors, including environmental requirements, nutrient uptake, photosynthesis, and metabolic pathways. The efficiency of photosynthesis in *Nannochloropsis* is influenced by the light intensity, quality, and photoperiod.

### 2.1. Environmental Factors

The cultivation of *Nannochloropsis* is highly dependent on various environmental factors. These factors play a crucial role in determining the growth rate, lipid content, and overall health of *Nannochloropsis* cultures. Understanding and managing these environmental conditions are essential for successful and efficient cultivation. Adequate light is essential for their growth.

As the light intensity increased from low to high levels, the growth rates of *N. oceanica* decreased in both autotrophic and mixotrophic cultures [21]. Comparatively, under light intensities of 40, 80, 120, and 160 µmol m^−2^ s^−1^, the biomass concentration and productivity of *N. gaditana* were highest at the light intensity of 120 µmol m^−2^ s^−1^ and a photoperiod comprising a 16 h light/8 h dark cycle [22]. Similarly, *Nannochloropsis* sp. demonstrated optimal growth with the maximum cell concentration under the conditions of a light intensity of 100 µmol m^−2^ s^−1^ and a photoperiod comprising a 18 h light/6 h dark cycle [23]. The light exposure significantly influences the production of compounds, particularly lipid synthesis, in *Nannochloropsis*. When subjected to light at an intensity of 500 µmol m^−2^ s^−1^, *N. oceanica* cells exhibited a 45% reduction in total protein content and a 38% decrease in sugar content, accompanied by a substantial 44% increase in total lipid content and more than 2-fold increases in β-carotene, zeaxanthin, astaxanthin, and canthaxanthin. The analysis using isobaric tags for relative and absolute quantification (iTRAQ) revealed that in carbon metabolism, glycolysis, the Calvin cycle, and the tricarboxylic acid (TCA) cycle were activated to channel more acetyl-CoA towards lipid and carotenoid biosynthesis [24]. The lower light intensity (30 and 50 µmol m^−2^ s^−1^), on the other hand, could promote the eicosapentaenoic acid (EPA) content in *N. oculata* to rise by 126% [25]. A similar trend is observed in *Nitzschia laevis*. Under the influence of elongases, C16:0-acyl carrier protein (ACP) can convert into C18:0, followed by the formation of C18:1 under the action of Δ9 desaturase. Additionally, C18:0 has the potential to generate C18:2 through stearoyl-ACP desaturase and Δ12-fatty acid desaturase (FAD). The up-regulation of these pathways suggests that low light conditions promote the biosynthesis of C18:1 and C18:2, which is beneficial for EPA biosynthesis [26]. Light wavelengths can influence the growth physiology of microalgal cells as well. It has been reported that low-light red light-emitting diodes (LEDs) could lead to a higher cell density and more fatty acids [27]. Analyzing different light wavelengths from red, green, blue, and white LEDs, it was observed that under blue light, *Nannochloropsis* sp. in both phototrophic and mixotrophic cultures exhibited the highest growth rates, with the fatty acid content reaching 20.45% and 15.11%, respectively [28]. The establishment of light–dark cycles contributes to the enhancement of cell photosynthetic efficiency; continuous light illumination, one the other hand, can result in energy loss and a decrease in cell density [28]. A significant correlation between metabolic status and light absorption rate was observed by Matsui et al. [29].

Temperature is another significant element of *Nannochloropsis* growth. Study has shown that the optimal growth temperature for *N. oceanica* falls between 25 and 29 °C, while growth ceases completely at temperatures above 31 °C and below 9 °C [30]. The suboptimal temperatures (<20 °C) were investigated, and the lowest growth rate but highest quantity of EPA occurred at 5 °C for *N. salina* [31]. Similarly, low-temperature stress at 10 °C favored the accumulation of EPA in *N. oculata*, enabling the EPA content to reach 12.8 mg L^−1^, which was 158% more than that obtained under 25 °C conditions [25]. By contrast, high temperature promoted lipid accumulation, with *N. gaditana* reaching the highest lipid content at 30 °C within the examined temperature ranges [32]. A sequential treatment approach was employed for *N. oceanica*, involving the initial addition of bicarbonate as the carbon source, followed by the application of low temperature to facilitate the redirection of carbon flux towards PUFA synthesis [33]. A study utilized a dual strategy involving random mutagenesis and adaptive laboratory evolution to acquire thermotolerant strains of *N. oculata*. Metabolomics and lipidomics unveiled a reconfiguration of the central carbon metabolism and membrane lipid synthesis in these thermotolerant strains [34].

Furthermore, it is imperative to consider the impact of symbiotic bacteria and fungi on the *Nannochloropsis* culture process. Fulbright et al. [35], for instance, isolated *Bacillus pumilus* from a underperforming *N. salina* culture in a 200 L system, and demonstrated its inhibitory effect on cell growth. On the other hand, there are symbiotic organisms that can be beneficial to *Nannochloropsis* growth. Du et al. [36], for example, combined the marine alga *N. oceanica* with the oleaginous fungus *Mortierella elongata*, resulting in increased biomass and production of triacylglycerols (TAGs).

### 2.2. Nutritional Factors

To harness their substantial potential in enhancing biomass and lipid production, carbon sources are frequently employed as supplements during cultivation. These carbon sources can be categorized into inorganic carbon, such as carbon dioxide [37] and bicarbonate [38], as well as organic carbon, including acetate [39], glucose [40], and glycerol [41]. Notably, a synergistic effect can be achieved by combining organic carbon and inorganic carbon. For instance, the addition of glycerol and carbon dioxide in the culture medium of *N. oculata* resulted in a significant increase in biomass and lipid production [42]. Furthermore, the interaction of carbon and nitrogen is of paramount importance for algae growth. The presence of acetate can greatly reduce the toxicity of ammonium, allowing it to be used as a nitrogen source in *N. oculata*. With 1 mM of ammonium and 32 mM of acetate in the culture, biomass and lipid production could be increased 1.5-fold and 9.5-fold, respectively [43]. It is worth noting that the presence of carbon promotes nitrogen utilization, and vice versa. When nitrate was introduced, glycerol consumption in *N. salina* was observed to shift from 60% to 79% [44].

Nitrogen and phosphorus comprise two of the most limiting factors in medium composition, and their deprivation often leads to lipid overproduction. Nitrogen availability is crucial for cell metabolism and the physiological and biochemical state in algae [45]. Phosphorus, on the other hand, is a component of several essential molecules that play a vital role in lipid turnover and remodeling [46]. The luxury uptake of phosphorus can act as a regulator of gene expression and an energy source [47]. The simultaneous occurrence of phosphorus stress and nitrogen stress has garnered interest. When the N:P supply ratio is raised to at least 32:1, phosphorus usage can be halved without harming the biomass productivity [48].

The presence of certain metal ions at relatively low concentrations is crucial for regulating cellular activities and plays important roles in growth and oil metabolism; these ions include Fe^2+^. Increasing the concentration of Fe^2+^ to 0.5 mmol L^−1^ results in a significant increase in PUFAs as a proportion of the total fatty acids in *Nannochloropsis* [49]. The optimal salinity range for *Nannochloropsis* growth and EPA accumulation falls between 27% and 29% [50]. Within a certain range, increasing the carbon dioxide concentration significantly boosts the EPA content in *Nannochloropsis* [51].

Moreover, combined factors have the potential to perform better than individual ones in lipid production. For example, the combination of salinity and light intensity could promote increased dry weight and lipid production in *Nannochloropsis* [52]. With combined nitrogen deprivation and high light exposure, *N. oculata* showed enhanced TAG accumulation compared to either nitrogen deprivation or high light exposure alone [53].

## 3. Biosynthesis of Lipids and High-Value Products by *Nannochloropsis*

### 3.1. TAG Biosynthesis

**Figure 1:**
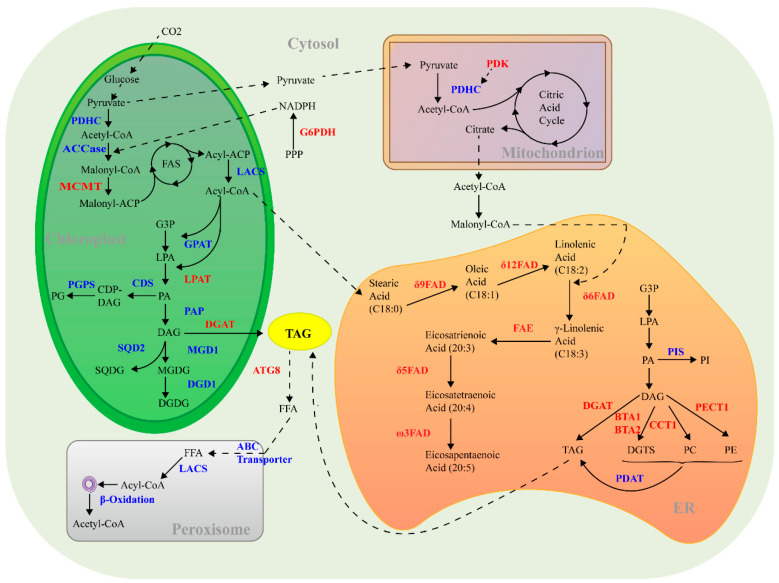
Reactions known or thought to be involved in lipid metabolism in *Nannocloropsis*. Enzymes are in bold. Characterized enzymes are highlighted in red, while those that have not yet been characterized are shown in blue. Abbreviations: ABC, ATP-binding cassette; ACCase, acetyl-CoA carboxylase; ATG8, ATG autophagy-related protein 8; BTA, betaine lipid synthase; CCT1, CTP:phosphocholine cytidylyltransferase 1; CDS, mitochondrial half-size ABC transporter; DGAT, diacylglycerol acyltransferase; DGD1, digalactosyldiacylglycerol synthase; FAD, fatty acid desaturase; FAS, fatty acid synthase; FAE, fatty acid elongase; GPAT, glycerol 3-phosphate acyltransferase; G6PDH, glucose-6-phosphate dehydrogenase; LACS, long-chain acyl-CoA synthetase; LPAT, lysophosphatidic acid acyltransferase; MCMT, malonyl-CoA:acyl carrier protein malonyltransferase; MGD1, monogalactosyldiacylglycerol synthase; PAP, phosphatidic acid phosphatase; PDAT, phospholipid:diacylglycerol acyltransferase; PDHC, pyruvate dehydrogenase complex; PDK, pyruvate dehydrogenase kinase; PECT1, CTP:phosphoethanolamine cytidylyltransferase 1; PGPS, phosphatidylglycerolphosphate synthase; PIS1, phosphatidylinositol synthase 1; SQD1, UDP-sulfoquinovose synthase 1; ACP, acyl carrier protein; CoA, coenzyme A; CDP, cytidine 50-diphosphate; DAG, diacylglycerol; DGDG, digalactosyldiacylglycerol; DGTS, diacylglycerol-N,N,N-trimethylhomoserine; ER, endoplasmic reticulum; FAT, fatty acyl-ACP thioesterase; FFA, free fatty acid; G3P, glycerol 3-phosphate; LPA, lysophosphatidic acid; MGDG, monogalactosyldiacylglycerol; NADPH, nicotinamide adenine dinucleotide phosphate; PA, phosphatidic acid; PC, phosphatidylcholine; PE, phosphatidylethanolamine; PG, phosphatidylglycerol; PI, phosphatidylinositol; SQDG, sulfoquinovosyldiacylglycerol; TAG, triacylglycerol.

TAGs are the main storage lipids in eukaryotic cells and have been widely used for foods, light industrial products, and biofuels. In the biofuel sector, TAGs are harnessed as a source of fatty acids for the transesterification process, which converts them into biodiesel. This chemical transformation involves reacting TAGs with an alcohol, typically methanol or ethanol, in the presence of a catalyst, resulting in the production of biodiesel (fatty acid methyl or ethyl esters) and glycerol as a byproduct. The renewable and sustainable nature of TAGs, especially those derived from sources like microalgae, makes them highly desirable for biofuels [54]. It is crucial to understand the pathways and key enzymes involved in TAG biosynthesis, as outlined in Figure 1.

Acetyl-CoA serves as the crucial carbon precursor for de novo fatty acid biosynthesis and can be derived from the conversion of pyruvate [55]. This conversion is negatively regulated by the enzyme pyruvate dehydrogenase kinase (PDK). Attenuating PDK has the potential to enhance TAG accumulation without impairing cell growth for *N. salina* [56]. Acetyl-CoA carboxylase (ACCase) is involved in the first step of de novo fatty acid biosynthesis, converting acetyl-CoA to malonyl CoA-ACP; *N. oceanica* is predicted to include multiple isoforms of ACCase [55]. As a key enzyme in the type II fatty acid synthesis (FAS II) pathway, malonyl CoA-ACP transacylase (MCAT) has been found to contribute to the increased lipid production and cell growth in *N. oceanica* [57]. The synthesized acyl-ACPs are either utilized in the chloroplast or released as free fatty acids, which are transported to the endoplasmic reticulum (ER) via transport proteins and long-chain acyl-CoA synthetase (LACS) [58]. In Arabidopsis, the half-size ATP-binding cassette (ABC) transporter subfamily A (ABCA) located in the ER is responsible for transporting acyl-CoA to the ER [59]. In *Nannochloropsis*, LACSs are believed to play a critical role in activating and facilitating the formation of acyl-CoA, which is essential for TAG synthesis, lipid elongation, and β-oxidation [60]. Transporter proteins are also predicted to exist in *Nannochloropsis* but have yet to be functionally identified [55].

There are multiple pathways towards TAG assembly. The Kennedy pathway serves as the primary TAG assembly pathway, comprising four enzymes of glycerol 3-phosphate acyltransferase (GPAT), lysophosphatidic acid acyltransferase (LPAT), phosphatidic acid phosphatase (PAP), and DGAT [61]. GPAT, the initial enzyme in the Kennedy pathway, catalyzes the transacylation of glycerol-3-phosphate (G3P) at the *sn*-1 position to lysophosphatidic acid (LPA). There are two GPAT isoforms in *N. oceanica*; the efforts to generate a disruption mutant of each GPAT have failed, indicating that both are essential for the alga [62]. Moreover, the overexpression of the ER-located GPAT in *N. oceanica* can lead to an increase in non-polar lipids [63]. In *N. oceanica*, four LPATs (LPAT1-LPAT4) have been identified, and they catalyze the transacylation of LPA at the *sn*-2 position to form phosphatidic acid (PA), with a preference for C16:0-CoA over C18:0-CoA as the acyl donor [62], similar to LPATs from the green alga *Chlamydomonas reinhardtii* [58]. The dephosphorylation of PA at the *sn*-3 position yields diacylglycerol (DAG), the precursor of TAG, which is catalyzed by PAP. It has been reported in *N. oceanica* that four putative cytoplasmic PAP2 genes are stimulated by TAG-induction conditions, indicative of their roles in TAG biosynthesis [55].

The final step in TAG assembly involves the transacylation of DAG to TAG, catalyzed by DGAT. In *N. oceanica*, 13 putative homologs of DGAT have been identified, including 2 DGAT1 genes (DGAT1A-B) and 11 DGAT2 genes (DGAT2A-K, also known as DGTT1-11) [17]. Among these, seven DGAT genes are upregulated under nitrogen-depleted conditions, including DGAT1A and six DGAT2 genes [55]. DGAT1A prefers C16 and C18 saturated/monounsaturated fatty acids as substrates, and its overexpression can result in a ~39% increase in the TAG content of *N. oceanica* under nitrogen deprivation [64]. As for DGAT2 genes, they have been reported to show different preferences on the substrates of acyl-CoA and DAG, play roles in TAG synthesis, and have great potential in the manipulation of *N. oceanica* for enhancing TAG levels and/or modifying fatty acid compositions [65,66,67,68,69].

Another well-characterized pathway for TAG synthesis in microalgae is acyl-independent and is mediated by phospholipid:diacylglycerol acyltransferase (PDAT). This enzyme transfers an acyl group from the *sn*-2 position of phospholipids (PLs) to form TAG [70]. Similar to in *C. reinhardtii*, PDAT in *N. oceanica* functions parallel to the DGAT-mediated pathway for TAG biosynthesis and shows preferences for the acyl donors [71,72]. It is worth mentioning that *N. oceanica* PDAT contributes more to the stress-associated TAG synthesis than to the basal TAG synthesis, seemingly distinct from *C. reinhardtii* PDAT [71].

### 3.2. EPA Synthesis

EPA, a ω3 long-chain polyunsaturated fatty acid (LC-PUFA), has long been recognized for its potential in preventing and treating human diseases [73]. *Nannochloropsis* is capable of producing EPA, and its biosynthesis pathway, starting from stearic acid (C18:0), involves a series of fatty acid desaturates (Δ9-FAD, Δ12-FAD, Δ6-FAD, Δ5-FAD, ω3-FAD) and a fatty acid elongase (Δ6-FAE) (Figure 1). These enzymes from *Nannochloropsis* have been functionally characterized to varying extents, via heterologous expression in the yeast *Saccharomyces cerevisiae*, overexpression, and/or knockdown [74,75,76,77,78,79]. It is believed that *N. oceanica* utilizes the ω6 pathway towards EPA biosynthesis, namely, from C18:0 via the intermediates C18:1Δ^9^, C18:2Δ^9,12^, C18:3Δ^6,9,12^, C20:3Δ^8,11,14^, and C20:4Δ^5,8,11,14^ [74,75]. By contrast, the diatom alga *Phaeodactylum tricornutum* employs both the ω6 and ω3 pathways for EPA biosynthesis [80], while *Thalassiosira pseudonana* likely uses only the ω3 pathway to produce EPA [81]. Additionally, an elongase (Δ0-ELO) that elongates C16:0 to C18:0 is involved in EPA biosynthesis, as its knockout disruption leads to a decreased EPA level in *N. gaditana* [82]. The overexpression of this elongase in *N. oceanica*, on the other hand, benefits EPA accumulation in TAG [69]. Understanding the key enzymes involved in the EPA synthesis pathway and their regulation is crucial for the engineering of EPA content and/or distribution in *Nannochloropsis* and also provides insights into manipulating other organisms for EPA biosynthesis.

It is generally accepted that EPA biosynthesis in *Nannochloropsis* occurs in the ER, where the key desaturases and elongases are predicted to be located. This is further confirmed via the ER subcellular localization experiment for Δ6-FAE, a key enzyme in EPA biosynthesis in *N. oceanica* [75]. It should be noted that in *Nannochloropsis*, similar to in diatoms, EPA is mainly distributed in the chloroplast membrane lipids [69], raising the question that how EPA is channeled from the ER into chloroplasts. While there have been several studies dealing with this, it remains largely unknown [83,84]. Additionally, EPA in chloroplast membrane lipids can be transferred to neutral lipids (NLs) like TAG during nitrogen deprivation, as demonstrated through ^13^C isotopic labeling in the microalgae *N. gaditana* [85]. The abundance of EPA in TAG, nevertheless, is relatively low for *Nannochloropsis* [69]. *Nannochloropsis* also produces long-chain hydroxy fatty acids (LCHFAs), long-chain alkenols (LCAs), and long-chain alkyl diols (LCDs); some enzymes involved in the biosynthesis of these compounds have been identified, including polyketide synthases (PKSs) and 3-hydroxyacyl dehydratases (HADs) [86].

### 3.3. Carotenoid Biosynthesis

The *Nannochloropsis* chloroplast evolves from the secondary endosymbiosis and is surrounded by four membranes. Unlike green algae with chlorophylls *a* and *b* or diatoms with chlorophylls *a* and *c*, *Nannochloropsis* contains only chlorophyll *a* in its light-harvesting pigment–protein complexes (LHCs) [87]. The proteomic analysis identified 17 LHC-type proteins among the 21 known in the genus *Nannochloropsis*, including LHCR-type proteins (related to red algae LHCI), LHCV proteins, and others [88]. LHCV also refers to violaxanthin/vaucheriaxanthin chlorophyll protein (VCP), where the two central carotenoids are surrounded by five chlorophyll *a* molecules, preserving the LHC superfamily structure [89]. The energy transfer efficiency of the VCP complex, from carotenoids to chlorophyll a, can reach up to 90% [90]. The two carotenoids serve as the accessory pigments in light harvesting for *Nannochloropsis* [91]. β-carotene is another prominent pigment in *Nannochloropsis*. Other carotenoids, such as neoxanthin, antheraxanthin, zeaxanthin, canthaxanthin, and astaxanthin, are also present in *Nannochloropsis* but at a low level [24,92]. Compared to green algae, *Nannochloropsis* lacks α-carotene and lutein [92].

**Figure 2:**
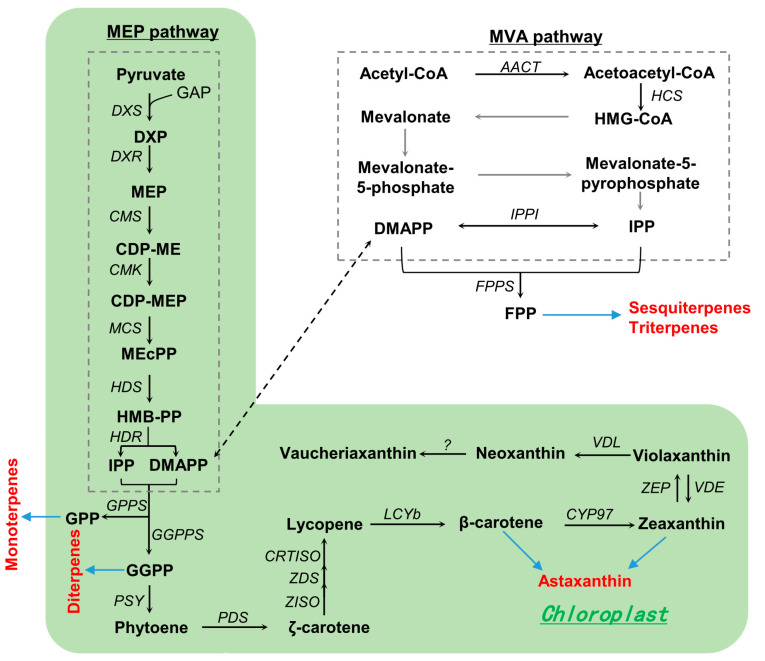
Proposed carotenoid biosynthetic pathways in *N. ocenica*. Abbreviations: AACT, acetoacetyl-CoA thiolase; CDP-ME, 4-diphosphocytidyl-2-C-methylerythritol; CDP-MEP, 4-diphosphocytidyl-2-C-methyl-D-erythritol 2-phosphate; CMK, 4-diphosphocytidyl-2-C-methyl-D-erythritol kinase; CMS, 2-C-methyl-D-erythritol 4-phosphate cytidylyltransferase; CRTISO, carotenoid isomerase; CYP97, cytochrome P450 beta-hydroxylase; DMAPP, dimethylallyl pyrophosphate; DXR, 1-deoxy-D-xylulose 5-phosphate reductoisomerase; DXP, 1-deoxy-D-xylulose 5-phosphate; DXS, 1-deoxy-D-xylulose 5-phosphate synthase; GAP, glyceraldehyde 3-phosphate; GGPP, geranylgeranyl diphosphate; GGPPS, geranylgeranyl diphosphate synthase; HCS, hydroxymethylglutaryl-CoA synthase; HDR, 4-hydroxy-3-methylbut-2-en-1-yl diphosphate reductase; HDS, 4-hydroxy-3-methylbut-2-en-1-yl diphosphate synthase; HMG-CoA, 3-hydroxy-3-methylglutaryl-CoA; HMB-PP, (E)-4-Hydroxy-3-methyl-but-2-enyl pyrophosphate; IPP, isopentenyl pyrophosphate; IPPI, isopentenyl-diphosphate Delta-isomerase; LCYB, lycopene beta cyclase; MCS, 2-C-methyl-D-erythritol 2,4-cyclodiphosphate synthase; mEcPP, 2-C-methyl-D-erythritol 2,4-cyclodiphosphate; MEP, 2-C-methylerythritol 4-phosphate; PDS, phytoene desaturase; PSY, phytoene synthase; VDE, violaxanthin de-epoxidase; VDL, violaxanthin-de-epoxidase-like enzyme; ZDS, zeta-carotene desaturase; ZEP, zeaxanthin epoxidase; ZISO, zeta-carotene isomerase; ?, unknown enzyme.

The carotenoid composition of *Nannochloropsis* can be substantially impacted by nutritional and environmental factors, with *N. oceanica* being the most well studied. Under high light illumination, *N. oceanica* shows a reduction in chlorophyll *a* and most carotenoids (e.g., violaxanthin, vaucheriaxanthin, β-carotene, and neoxanthin), accompanied by an increase in zeaxanthin [24,92,93]. Based on the carotenoid profiles and in silico analysis of carotenogenic genes, carotenoid biosynthetic pathways for *N. oceanica* have been proposed [92], including the production of isopentenyl diphosphate (IPP) and dimethylallyl diphosphate (DMAPP) via the 2-C-methylerythritol 4-phosphate (MEP) pathway, β-carotene biosynthesis from IPP/DMAPP, and β-carotene-derived xanthophyll biosynthesis (Figure 2). Briefly, IPP/DMAPP molecules produced in the MEP pathway are condensed to the 20-carbon geranylgeranyl diphosphate (GGPP) and then to the 40-carbon phytoene through the action of GGPP synthase (GGPPS) and phytoene synthase (PYS), respectively. Phytoene is then desaturated, isomerized, and cyclized to β-carotene, mediated by phytoene desaturase (PDS), ζ-carotene desaturase (ZDS), carotenoid isomerase (CRTISO), and lycopene β-cyclase (LCYB). β-carotene, catalyzed by the heme-containing cytochrome P450 enzymes, is hydroxylated to zeaxanthin, which can be epoxidated to violaxanthin and de-epoxidated back to zeaxanthin, by the enzyme pair zeaxanthin epoxidase (ZEP) and violaxanthin de-epoxidase (VDE). Violaxanthin can be further converted to neoxanthin by a violaxanthin-de-epoxidase-like enzyme (VDL) and then to vaucheriaxanthin via yet-to-be-identified enzymes.

So far, the functional characterization of carotenogenic enzymes for *N. oceanica* remains limited. It has been reported that PDS overexpression has little effect on carotenoid profiles, while LCYB overexpression benefits the accumulation of carotenoids, particularly β-carotene; in this context, LCYB, rather than PDS, mediates a rate-limiting step for carotenoid biosynthesis in *N. oceanica* [92]. There are two phylogenetically distant ZEP isoforms in *N. oceanica*, namely, ZEP1 and ZEP2. The functional roles of the two ZEPs have been revealed in a recent study, which supports the hypothesis that both ZEP1 and ZEP2, localized in the chloroplast, overlap in epoxidating zeaxanthin to violaxanthin for the light-dependent growth of *N. oceanica* [93]. It is worth noting that ZEP1 appears to be more functional than NoZEP2 [93]. *N. oceanica* harbors one VDE; deactivating this enzyme via insertional disruption can attenuate zeaxanthin accumulation substantially in response to high light and thus leads to growth impairment [94]. Likely, zeaxanthin contributes to the induction of the non-photochemical quenching and scavenging of reactive oxygen species, thus playing an essential role in protecting the alga from excess light. While violaxanthin is essential for *N. oceanica*, neoxanthin and vaucheriaxanthin are not, as a disruption to VDL that leads to the abolishment of the two carotenoids is viable [95]. As expected, the VDL-deficient mutant accumulates more violaxanthin [95].

Violaxanthin exhibits significant antioxidative capacity and anti-inflammatory activity against cancer cells [96]. A previous study has demonstrated that extracts from *Nannochloropsis*, primarily composed of violaxanthin, can protect against UV-B-induced damage [11]. In this context, the pursuit of violaxanthin enhancement in *Nannochloropsis* is an intriguing avenue of exploration. The overexpression of ZEPs can enable *N. oceanica* to synthesize more violaxanthin, yet the increase remains to be enlarged [93]. Although *N. oceanica* synthesizes astaxanthin, a valuable ketocarotenoid with broad applications [97], the level is very low and generally detectable under stress conditions [24]. The enzymes responsible for astaxanthin biosynthesis in the alga have not been identified. Given the relative abundance of β-carotene, the direct precursor of astaxanthin, introducing a strong β-carotenoid ketolase has the potential to allow *N. oceanica* for synthesizing astaxanthin in addition to EPA [98,99]. Considering the function of ZEP1/2 [93], suppressing its expression may reallocate carotenoid flux towards astaxanthin synthesis in the engineered *N. oceanica*. The accumulated astaxanthin may, in turn, endow the alga with an improved production performance under high light, as is the case for *C. reinhardtii* [100]. Moreover, allowing astaxanthin storage in the cytosolic lipid droplets, like in the astaxanthin-rich *Haematococcus pluvialis* [101], will be a promising strategy for a further increase in astaxanthin in the engineered *N. oceanica*.

### 3.4. Other High-Value Products

IPP/DMAPP has multiple destinations: (1) to farnesyl diphosphate (FPP) by FPP synthase (FPPS)—this can be used for the synthesis of sesquiterpenes (e.g., humulene, artemisinic acid, artemisinin) and triterpenes (e.g., dogfish, olefin, oleanolic acid, aromatic acid); (2) to form geranyl diphosphate (GPP) by GPP synthase (GPPS), which can serve as the precursor to monoterpenes (e.g., limonene, menthol); and (3) to GGPP, which can be used for synthesizing diterpenes (e.g., sclareol, taxadiene, paclitaxel) beyond carotenoids. *Nannochloropsis* has the potential to synthesize these valuable terpenoids via metabolic engineering as long as the corresponding enzymes can be functionally expressed in the alga (Figure 2). As a proof of concept, the biosynthesis of certain terpenoids has been achieved in the green alga *C. reinhardtii* via metabolic engineering [102,103,104].

*Nannochloropsis* can be employed as a potential host for the production of recombinant proteins. For example, *N. oceanica* has been genetically engineered to produce the surface protein VP2 of the infectious pancreatic necrosis virus, which causes high mortality rates in young salmonids [105]. *Nannochloropsis* is also a promising candidate for the production of marine antimicrobial peptides. These peptides have garnered attention due to their natural ability to combat various pathogens and microbes in the marine environment. The oral administration of antimicrobial peptide-producing *N. oculata* can enable substantial improvements in the survival rate of medaka after infection with *Vibrio parahaemolyticus* [106]. Moreover, the biosynthesis of a fish growth hormone has been achieved in *N. oculata*; this hormone can promote the growth of red tilapia larvae [107]. Overall, genetic engineering and synthetic biology hold the key to utilizing *Nannochloropsis* to produce targeted products.

## 4. Cultivation Strategy for *Nannochloropsis* Production

To optimize *Nannochloropsis* production, a well-thought-out cultivation strategy is essential. Nitrogen deprivation is a well-established strategy for inducing lipid accumulation in *Nannochloropsis*. When nutrients are limited, the algal cells tend to divert energy and carbon toward lipid biosynthesis. Nitrogen deprivation can trigger TAG accumulation in *N. gaditana*, accompanied by a reduction in polar lipids and the partial translocation of EPA from polar lipids to TAG [108]. A reduction in EPA content is also observed for *N. gaditana* responding to nitrogen deprivation, which is independent of light regimes and intensities [109]. There is a considerable decrease in chlorophyll content upon nitrogen deprivation, accompanied by changes in carotenoids [54].

Multi-stage cultivation represents an effective strategy for maximizing lipid production from *Nannochloropsis*. In the initial stage, *Nannochloropsis* is cultivated under nutrient-replete conditions to grow fast and achieve high cell densities. Following the growth phase, the culture is subjected to stress-inducing conditions. Common stressors include nutrient limitations, such as nitrogen and phosphorus deprivation. Stress conditions divert the energy and metabolic resources of the algal cells away from growth to the synthesis of lipids. Such a two-stage cultivation approach can lead to more than a 2-fold increase in lipids for *Nannochloropsis* [110]. Another effort using a two-stage continuous cultivation approach for *N. gaditana*, which involved four photobioreactors connected in series, resulted in a remarkable 7.7-fold increase in biomass and a 46% increase in lipid productivity [111].

A fed-batch culture is a sophisticated strategy for production improvements. In this approach, nutrients are supplied incrementally during the growth phase, allowing for higher cell densities and enhanced productivity. This strategy has been employed for the cultivation of various *Nannochloropsis* species, allowing a considerably higher EPA abundance (based on total fatty acids) and yield in *N. oculata* [112] and greater biomass production and EPA yield in *N. oceanca* [69]. Implementing these cultivation strategies, alongside ongoing research and innovation, will play a pivotal role in realizing the full potential of *Nannochloropsis* for sustainable and economically viable production.

## 5. Genetic Tools for *Nannochloropsis* Manipulation

### 5.1. Genetic Resources

Several species of *Nannochloropsis* have been sequenced, with a nuclear genome size ~29 Mb nuclear genome that encodes a total of over 10,000 genes [17,113,114,115,116,117]. There are increasing number of transcriptomes regarding *Nannochloropsis* under different conditions [45,46,55,118,119,120,121,122,123,124]. These genome and transcriptome data help us to understand the biology of *Nannochloropsis* and its metabolic adaptability to respond to dynamic environmental changes, and, on the other hand, provide genetic resources for the genetic engineering of this organism.

### 5.2. Transformation Methods, Selectable Markers, and Reporters

*Nannochloropsis* species contain a rigid cell wall, which serves a barrier for the penetration of transgenes. The first successful nuclear transformation of *Nannochloropsis* was conducted in 2011 via electroporation with a very high voltage [125]. Since then, the electroporation-mediated transformation method has been established for various *Nannochloropsis* species [115]. Compared to linearized plasmids, the use of a PCR-amplified cassette can improve *Nannochloropsis*’ transformation efficiency [126]. The co-transformation of *Nannochloropsis* with two genes has also been achieved [126]. With the assistance of secondary metabolites from myxobacteria that help weaken the algal cell wall, a 2.7-fold increase in transformation efficiency can be reached for *N. salina* [127]. Other nuclear transformation methods besides electroporation have been developed as well, such as agrobacterium-mediated transformation for *Nannochloropsis* sp. [128] and particle bombardment for *N. oceanica* [129]. Moreover, an electroporation-mediated chloroplast transformation method has been developed, opening up possibilities for plastid genome editing in *Nannochloropsis* [130]. These diverse transformation techniques offer researchers tools to engineer *Nannochloropsis* for target applications.

Screening transformants of *Nannochloropsis*, regardless of the transformation methods, requires the assistance of proper selectable markers. The most widely used selectable marker is the bleomycin-resistant gene, which confers *Nannochloropsis* with resistance against zeocin [125]. Additional feasible markers for *Nannochloropsis* transformation include hygromycin-B phosphotransferase, blasticidin-S deaminase, aminoglycoside 3′-phosphotransferase, and nourseothricin acetyltransferase, which are resistant to hygromycin B, blasticidin, G418, and nourseothricin, respectively [15]. These antibiotic resistance genes are not desirable, as they have their limitations and face regulatory hurdles, particularly for food uses, driving the development of endogenous genes as alternative selectable markers. One promising candidate is the mutated *PDS* gene, which has been employed in several algae to confer resistance to herbicides [131,132,133,134]. While selectable markers facilitate the screening of transgene integration into the nuclear genome, reporters play a pivotal role in monitoring transgene expression at the protein level or subcellular localization when fused with transgenes. The workable reporters in *Nannochloropsis* include green fluorescent protein [135], sfCherry fluorescent protein [136], luciferases (Nlux, Flux) [74], and purple chromoprotein [137], among others.

To enable the efficient expression of transgenes in *Nannochloropsis*, regulatory elements are needed, for example, promoters, terminators, enhancers, and transit peptides. Generally, strong endogenous promoters are used for transgene expression in *Nannochloropsis*, including those from ubiquitin extension protein (UEP) [115,136], β-tubulin (β-tub) [56,138], lipid droplet surface protein (LDSP) [17,74], elongation factor (EF) [66,74], violaxanthin/chlorophyll a-binding protein 2 (VCP2) [64], and ribosomal subunits (Ribi) [74]. The frequently used terminators are those from LDSP, VCP1, and heat shock protein (HS) [15]. To facilitate the transcriptional expression of transgenes, a leader-enhancing sequence may be placed upstream of the transgene [65]. To guide the compartmentation of expressed proteins, transit peptides are needed, for example, the chloroplast transit peptides from glycine cleavage system protein L (GCSL) and VCP1 [135,139]. There is an increasing need for the characterization of more regulatory elements (e.g., inducible promoters, dose-dependent promoters) to meet the needs of diverse uses in the genetic engineering of *Nannochloropsis*.

### 5.3. Genetic Tools

The implementation of *Nannochloropsis* engineering for trait manipulation requires sophisticated genetic tools. Overexpression represents a frequently employed approach for the engineering of *Nannochloropsis*, which typically involves a single gene of interest. To achieve the expression of multiple genes, these are assembled in separate vectors for *Nannochloropsis* transformation [69,74]. This, however, requires the use of multiple antibiotic resistance markers. To reduce the use of antibiotic resistance markers, a high-capacity gene-stacking toolkit has recently been developed that takes advantage of the Gateway cloning technique and allows the easy assembly of multiple gene expression cassettes (with individual promoters and terminators) into a single vector for *N. oceanica* transformation [15]. The transgenes can also be assembled in a single vector, fused by self-cleaving 2A peptides, and have proved workable for multigene expression in *N. oceanica* [74] and *N. salina* [140]. This technique eliminates the use of multiple promoters and terminators and thus can reduce the size of vectors. Nevertheless, even with the optimization of 2A types, the cleavage rate of 2A-fused proteins is not high, reaching no more than 50% in *N. oceanica* [74]. To enable the synthetic biology of *Nannochloropsis,* a more sophisticated and standardized toolkit is needed. A modular cloning toolkit has been developed for the green alga *Chlamydomonas*; the toolkit is based on the Golden Gate cloning method with standard syntax and comprises over 100 openly distributed genetic parts [141]. This provides insights into the development of such a toolkit for *Nannochloropsis*.

Besides overexpression, suppression is a common employed strategy to understand gene function and can be achieved via knockdown or knockout. In *Nannochloropsis*, RNA interference (RNAi)-mediated gene knockdown has been proven feasible, with the knockdown efficiency reaching up to 80% [56,64,71,93,142,143]. As for gene knockout, the homologous recombination (HR) approach has long been applied to *Nannochloropsis*, with a starting effort in inactivating the nitrate reductase gene [125]. However, due to the low HR efficiency in *Nannochloropsis*, HR-mediated gene knockout has only been achieved by a limited number of studies [62,144]. The CRISPR/Cas system represents an emerging technology in gene editing and has been widely applied to various organisms. Using the nitrate reductase gene as the target, the CRISPR/Cas9-mediated gene editing of *Nannochloropsis* was first developed in *N. oceanica* but with a very low efficiency of less than 1% [138]. With the assistance of homologous DNA templates, the CRISPR/Cas9-mediated gene knockout efficiency in *Nannochloropsis* can be improved and varied depending on the target genes [145]. To eliminate the restriction of selectable markers, the combination of CRISPR/Cas9 with an inducible Cre recombinase has been introduced into *N. gaditana*; this can enable the removal of marker genes and thus the potentially unlimited stacking of gene knockouts in the alga [146]. As well as being integrated into the nuclear genome, the CRISPR/Cas9 in the plasmid with the presence of the CEN/ARS6 region from *S. cerevisiae* can be maintained as circular extrachromosal DNA in *N. oceanica*; once targeted mutations are generated, the episomal DNA can be removed in the absence of selection pressure, resulting in marker-free non-transgenic engineered strains [147]. Furthermore, Cas ribonucleoprotein can be delivered into *N. oceanica* via electroporation for gene editing, thus eliminating the involvement of the plasmid; the editing efficiency using the Cas12a from *Francisella novicida* can reach up to 90% [148]. With this Cas12a, an episomal plasmid-based CRISPR/Cas12a system has been developed, capable of performing multiplexed gene editing in *N. oceanica* [149]. Moreover, CRISPR/Cas9-based transcription interference and activation systems have also been established for *N. oceanica* [149,150].

## 6. Conclusions

*Nannochloropsis* harbors advantageous features and has attracted increasing interest from academic and commercial stakeholders. It has emerged as a focal point in synthetic biology due to its versatile genetic characteristics and potential applications in biofuel production, bioremediation, and the synthesis of valuable compounds. Yet there are many things to do to increase the production potential of *Nannochloropsis*. Firstly, the cultivation strategies for *Nannochloropsis* need to be upgraded, with an emphasis on large-scale production for various applications. The development of next-generation photobioreactors and corresponding cultivation strategies will be instrumental in achieving commercial-scale biorefining. Secondly, the application of advanced omics technologies will further our understanding of *Nannochloropsis*’ biology and metabolism, enabling more precise engineering for trait improvements. Thirdly, synthetic biology tools will continue to play a pivotal role in tailoring *Nannochloropsis*. This will depend on the development of versatile and robust toolkits. Learning from other algae such as *C. reinhardtii* and *P. tricornutum* would provide valuable insights and solutions for advancing the synthetic biology of *Nannochloropsis*. *Nannochloropsis*-based biorefineries hold great potential for the sustainable production of biofuels, high-value chemicals, and nutraceuticals. A key focus will be to optimize production processes to ensure economic viability and to meet the growing demand for renewable and environmentally friendly products. Navigating the potential applications of *Nannochloropsis* in synthetic biology is not without its set of challenges. One of the primary hurdles lies in achieving a meticulous level of control over the genetic modifications made to this microalgae genus. Ensuring the stability and predictability of the outcomes is essential for the successful integration of *Nannochloropsis*-based technologies into industrial processes. The precision required in genetic manipulation becomes increasingly critical as efforts are made to scale up production and consistently meet the demands of various industries.

In conclusion, *Nannochloropsis* represents a promising light-driven cell factory in the realm of synthetic biology. The inherent ability to synthesize abundant lipids and valuable pigments makes *Nannochloropsis* an ideal candidate for the sustainable production of biofuels and high-value compounds. Through the concerted efforts of researchers and the continued development of synthetic biology tools, we anticipate substantial advancements in strain engineering, large-scale production, and biorefining. With ongoing research and innovation, *Nannochloropsis* is set to contribute to shaping a more sustainable and greener future.

## Data Availability

The data presented in this study are available on request from the corresponding author.
